# Primary Ewing’s sarcoma in a small intestine – a case report and review of the literature

**DOI:** 10.1186/s12893-020-00774-z

**Published:** 2020-05-25

**Authors:** Andrej Kolosov, Audrius Dulskas, Kastytis Pauza, Veslava Selichova, Dmitrij Seinin, Eugenijus Stratilatovas

**Affiliations:** 1 Vilnius University, Faculty of Medicine, Vilnius, Lithuania; 2 National Cancer Institute, Vilnius, Lithuania

**Keywords:** Ewing’s sarcoma, Extraosseus sarcoma, Primitive neuroectodermal tumor, Small bowel sarcoma, ERG-FUS translocation

## Abstract

**Background:**

Ewing’s sarcoma usually presents in paediatric patients with its primary location being bone tissue. Nevertheless, we present such an adult case which arises from the small intestine. We registered thirty one cases of such origin published so far excluding ours.

**Case presentation:**

We report a case of 30 year old female who was admitted due to the persistent anaemia. Whole body computed tomography scan revealed abdominal mass in her left upper abdominal compartment. Surgery on the mass originating from jejunum was performed, although due to extremely complicated postoperative period and rapid dissemination no additional therapy had been performed. The tumour was positive for CD99, ERG, CD56, Synaptophysin, PanCK, Cam5.2.

**Conclusion:**

Extraosseus Ewing’s sarcoma is extremely rare entity, with poor prognosis.

## Background

Ewing’s sarcoma was first identified by A.P. Stout in 1918 [1]. Genetically Ewing’s sarcoma is determined by recurrent balanced translocations involving the EWSR1 gene on chromosome 22 and members of the E-twenty six (ETS) family of transcription factor genes [2–4]. It includes extra-osseous (also called extra-skeletal) Ewing’s sarcoma, peripheral neuroepithelioma, Askin’s tumour (tumour of chest wall), and peripheral neuroblastoma [4, 5]. Primitive neuroectodermal tumours (PNET) are also referred as Ewing‘s sarcoma [6]. Histologically they are presented as small round cell tumours which originate from the bone and soft tissue [7]. Nevertheless they can be found in most organs: chest (44%), retroperitoneum and pelvis (26%), extremities (20%), head and neck (6%), kidneys, oesophagus, ovaries, prostate [5–10] and are especially rare in small bowel [7]. To make matters clearer, they are divided into pPNET (peripheral primitive neuroectodermal tumours), CNS primitive neuroectodermal tumours (PNETs) and neuroblastoma [11]. While microscoping pPNET looks almost identical to the tumours originating from the bone [12] which makes them hard to differentiate especially when it originates near the bone. In all cases Ewing’s sarcoma metastasises rapidly disregarding its origin [13]. It usually occurs in young patients, with paediatric patients being a second most common tumour in this age group [5, 12–15]. In older patients survival rate is lower and there are more cases with metastatic spread or extra skeletal tumours [3]. The common sites of Ewing’s sarcoma recurrence are the soft tissue of the lower extremities, paravertebral region, and pelvis [14]. Five year overall survival rate is 44% (ranging 20–60%) [16].

Up to date only 31 cases of extraosseus Ewing’s sarcoma arising in a small intestine are reported in the literature (Table 1).

**Table 1:** Clinical characteristics and outcomes of patients with intestinal Ewing sarcoma

Author, year	Sex	Age, y	Part of small intestine	Treatment	Survival, months	MTS on diagnosis
Horie, 2000 [15]	M	40	Mesentery of jejunum	Surgery - > systemic therapy	5	N/A
Shek, 2001 [9]	F	9	Mesentery of the small bowel	Surgery- > systemic therapy - > surgery- > systemic therapy	25	pelvis
Saranganathan, 2001 [17]	M	13	Jejunum	Surgery	12+	Absent
Balasubramanian, 2002 [16]	F	53	Mesentery of ileum	Surgery	N/A	N/A
Graham, 2002 [2]	M	14	Distal ileum	Surgery- > systemic therapy	52+	N/A
Boehm, 2003 [18]	M	18	Ileum	Surgery- > systemic therapy - > regression - > systemic therapy, autologous cell Tx	+	peritoneum
Bala, 2006 [8]	F	57	Terminal ileum	Surgery- > systemic therapy	8+	N/A
Batziou, 2006 [12]	M	66	Small intestine	2x surgery - > 4 systemic therapy	48+	N/A
Kim, 2007 [14]	M	63	Terminal ileum and jejunum	Surgery- > systemic therapy	N/A	Regional l/n
Sethi, 2007	M	44	Terminal ileum	Surgery- > 6 courses systemic therapy	13	N/A
Rodarte-Shade, 2012 [19]	M	32	Ileum	Surgery- > systemic therapy	6+	Absent
Vignali, 2012 [20]	F	15	Terminal ileum, mesentery	Surgery- > systemic therapy	N/A	N/A
Kim, 2013 [4]	M	23	Mesentery of jejunum	Surgery- > systemic therapy - > surgery	N/A	Porta hepatis, l/n
Rachan Shetty, 2014	F	24	Ileum	Surgery - > systemic therapy	15+	N/A
Milione, 2014 [3]	M	18	Ileum	Core biopsy	8	Liver
Milione, 2014 [3]	M	20	Ileum	Core biopsy	28	Liver
Milione, 2014 [3]	M	42	Ileum	Surgery - > systemic therapy	11	Absent
Milione, 2014 [3]	M	45	Ileum	Surgery - > systemic therapy	13	Absent
Milione, 2014 [3]	F	15	Ileum	Surgery - > systemic therapy + radiotherapy	28	Absent
Milione, 2014 [3]	M	57	Ileum	Surgery	+	Absent
Milione, 2014 [3]	F	28	Ileum	Surgery	204+	Liver
Peng, 2015 [13]	M	59	Mesentery of terminal ileum	Surgery - > no adjuvant therapy	N/A	N/A
Peng, 2015 [13]	M	22	Ileum	Surgery - > no adjuvant therapy	N/A	Liver
Peng, 2015 [13]	F	36	Mesentery of ileum	Surgery - > 8 cycles systemic therapy	34	N/A
Padma, 2015 [5]	F	22	Distal jejunum	Surgery	N/A	Absent
Liu, 2015	M	15	Mesentery of jejunum	Surgery	7	N/A
Li, 2017 [7]	F	16	Ileum	Surgery	N/A	N/A
Kim, 2017 [10]	F	9	Jejunum	Systemic therapy - > surgery - > adjuvant therapy	N/A	peritoneum
Liao, 2018 [21]	F	25	Ileum	Surgery	+	N/A
Cantu, 2019 [22]	F	67	Jejunum	Surgery	3+	No
Yagnik, 2019 [11]	M	42	Jejunum	Surgery - > systemic therapy	+	N/A
Our case, 2020	F	30	Jejunum	Surgery	2	No

Tx – Transplant

N/A – Not available

L/n – Lymph nodes

+ − No information of death upon the last follow up

Here we present a case of Ewing’s sarcoma in a small intestine with an extremely rapid dissemination and unfortunate ending.

## Case presentation

**Fig. 1:**
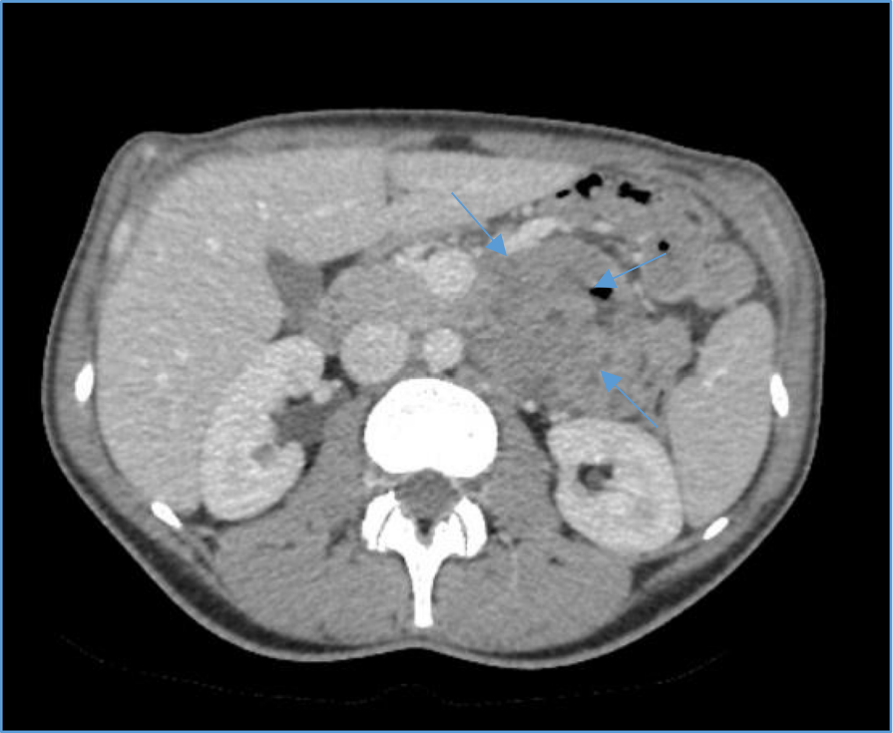
Computed tomography scan of the abdomen. Axial transverse view of 4.7 × 6.2 × 6.5 cm non-homogenic tumour (blue arrows) with a cystic component contacting with an inferior mesenteric vein

**Fig. 2:**
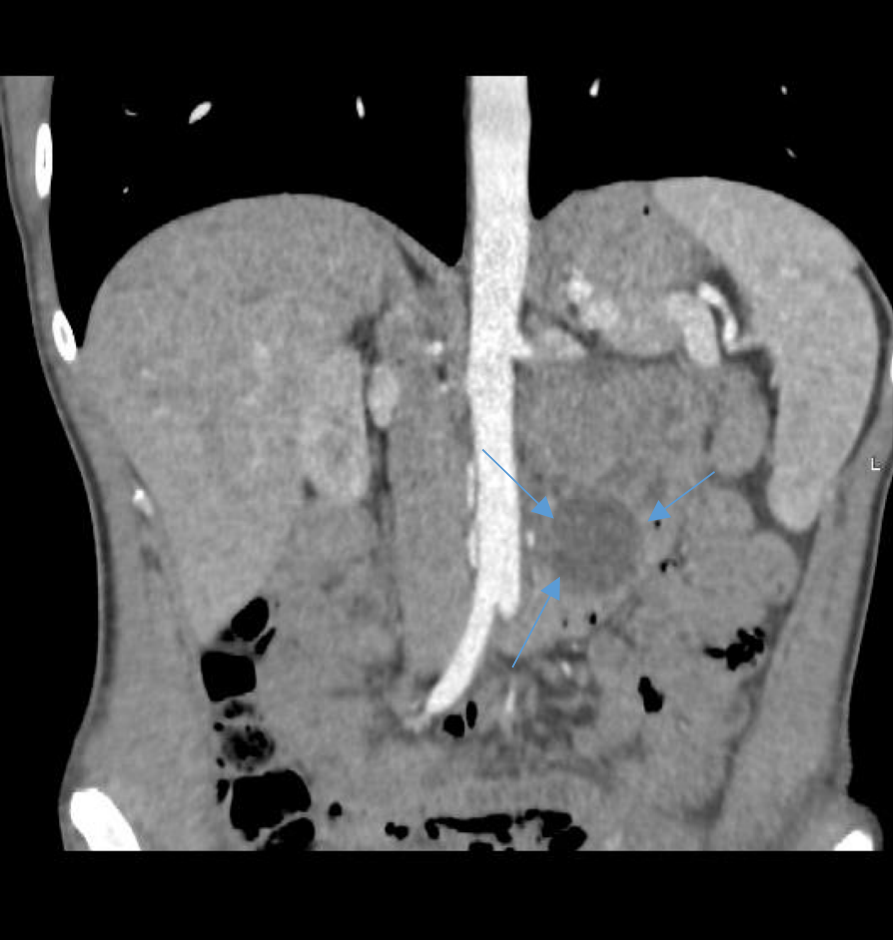
Computed tomography scan of the abdomen. Coronal view – 4.7 × 6.2 × 6.5 cm non-homogenic tumour with a cystic component contacting with an inferior mesenteric vein (blue arrows)

Here we present a 30 year old female admitted to local hospital complaining of mild general weakness lasting for four months. Full blood count showed severe anaemia of 54 g/l and a mild gastric wall inflammation was seen on gastroscopy. Blood transfusion in therapeutic ward was then performed. Whole body computed tomography (CT) scan discovered 4.7 × 6.2 × 6.5 cm non-homogenic tumour with a cystic component in a left upper abdominal compartment. Tumour had a close contact with an inferior mesenteric vein (IMV) at the long segment, near the entrance to the superior mesenteric vein (SMV) (Type A/B) without overgrowing any another surrounding organ. Gastrointestinal stromal tumour (GIST) was suspected (Figs. 1 and 2). Patient was transferred to our cancer institute and after multidisciplinary team discussion she was elected for a surgery – removal of the GIST of the jejunum. The patient had no previous medical history of other diseases, surgeries or allergies. Her initial blood test showed haemoglobin of 74.6 g/l, so additional blood transfusion was performed.

**Fig. 3:**
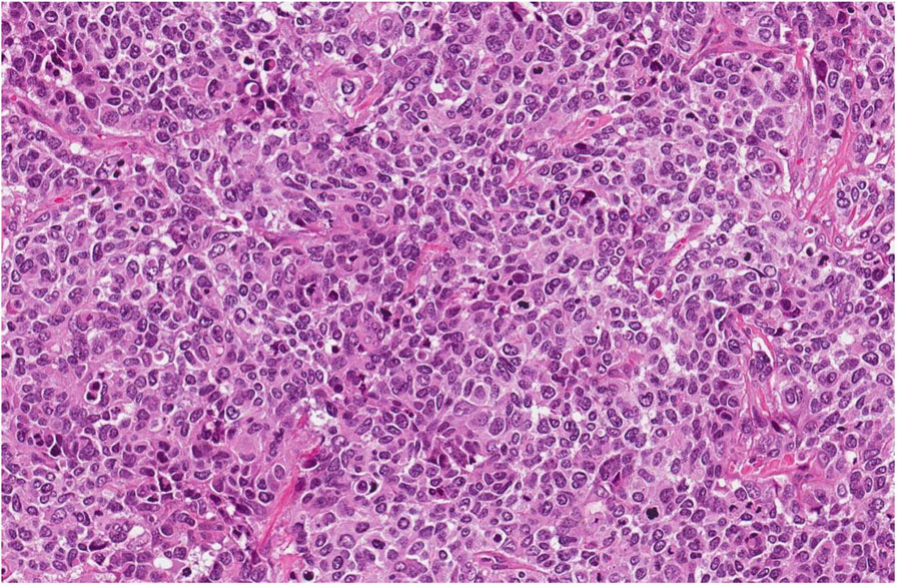
Tumour is composed of sheets of mildly to moderately atypical cells with round or oval nuclei and prominent nucleoli. There were up to 60 mitoses per 10 HPF, HE 20x

**Fig. 4:**
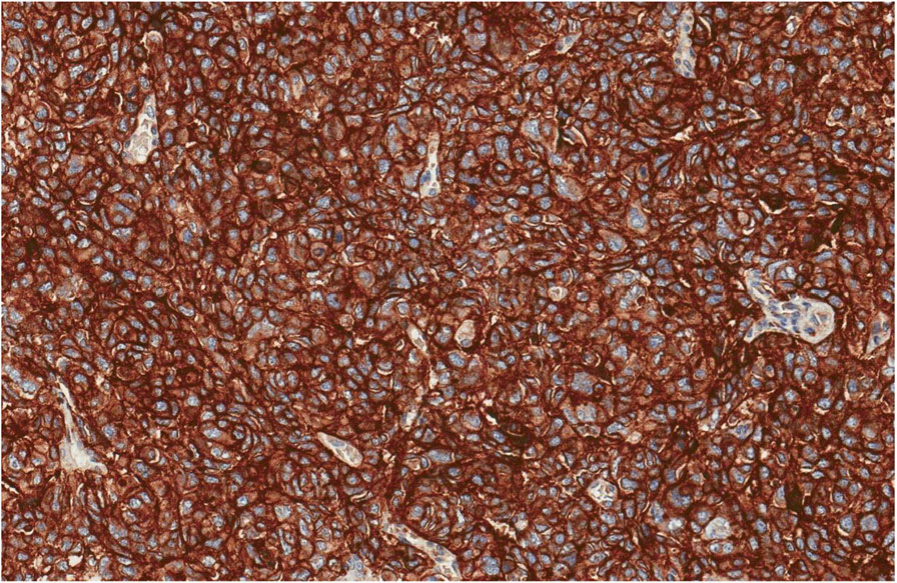
Tumour showed diffuse membranous positivity for CD99

**Fig. 5:**
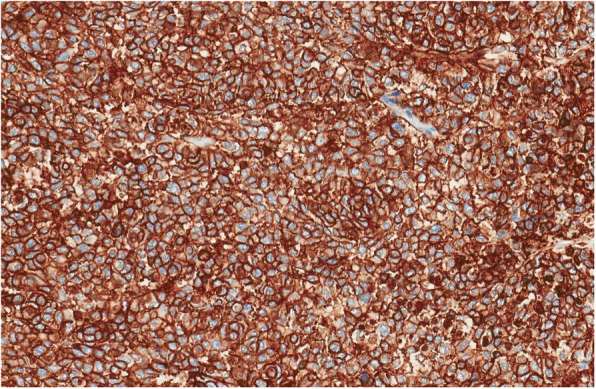
Tumour showed diffuse membranous positivity CD56

**Fig. 6:**
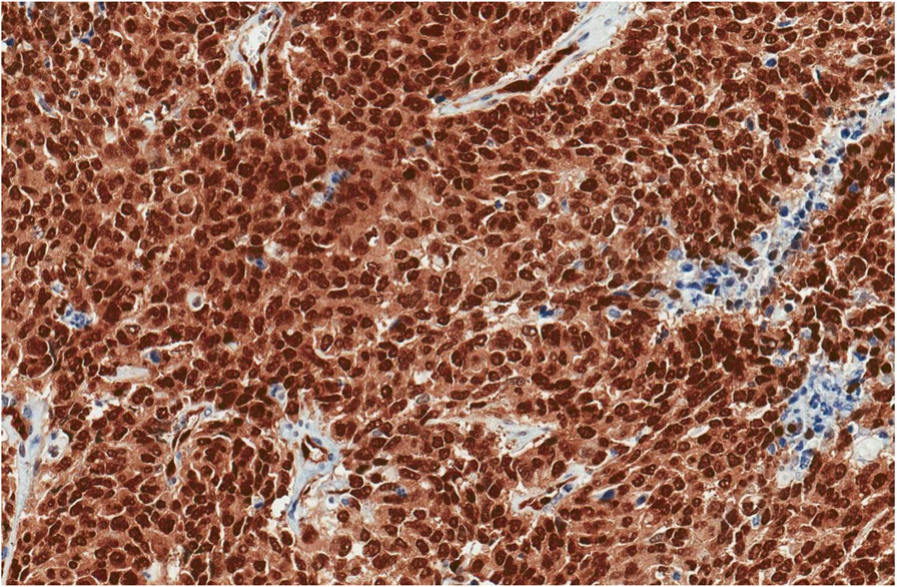
Diffuse nuclear positivity for ERG

**Fig. 7:**
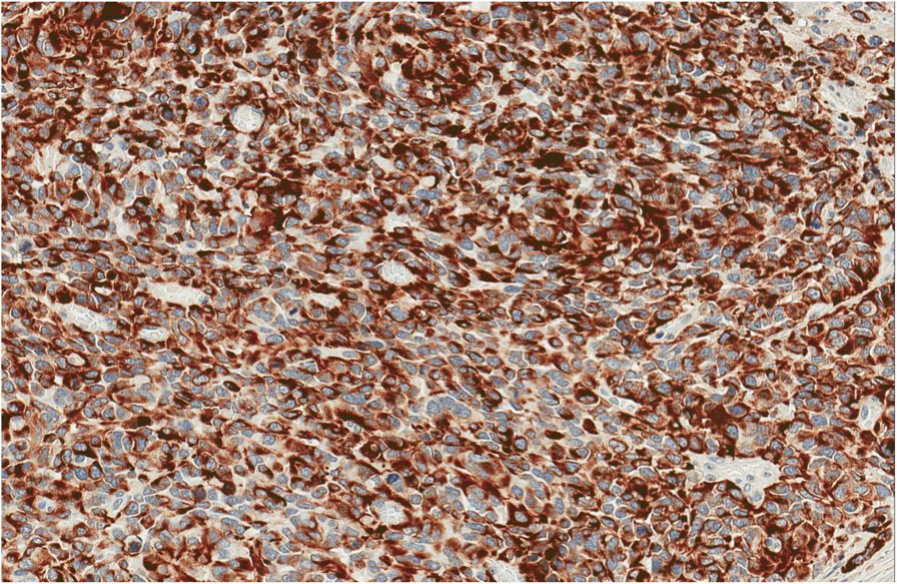
Focal immunoreactions for Cam5.2

**Fig. 8:**
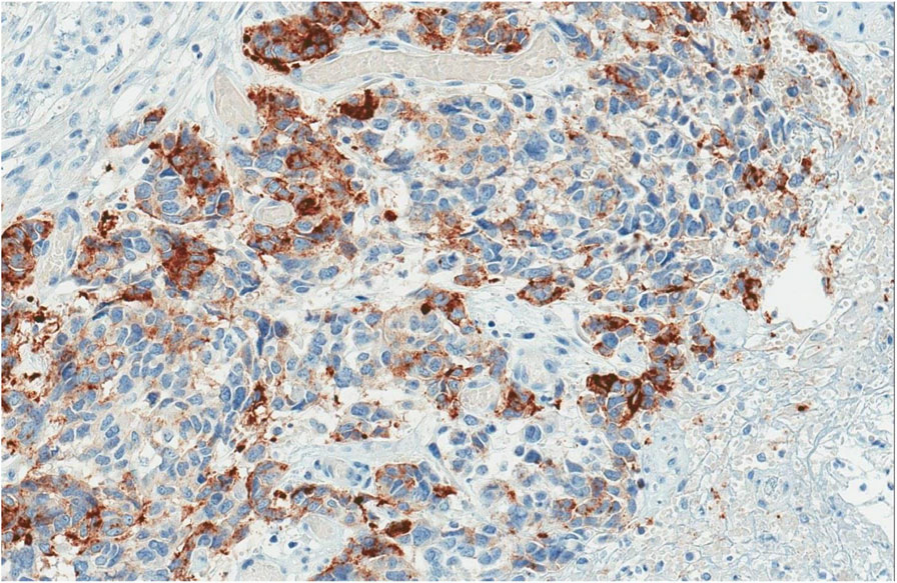
Focal immunoreactions for Synaptophysin

On laparotomy the tumour involving the first loop of the small intestine (30 cm from the *Treitz* ligament), a part of uncinated process of the pancreas, IMV and inferior mesenteric artery (IMA) was found. Enlarged small intestine lymph nodes were also seen. Resection of 50 cm of small intestine with the tumour was performed. Two layer end-to-end entero-entero anastomosis was performed and enteral feeding tube was inserted below the anastomosis. Early postoperative course was uneventful. On postoperative day (POD) five the blood appeared through the drains – emergency surgery was initiated. Massive bleeding from superior mesenteric artery (SMA) was found and the arterial defect was sutured. The cyanotic small intestines were noticed and a partial occlusion of SMA was suspected. Aorto-mesenteric shunt using saphenous vein was performed and the small intestine regained its blood supply. Two days later anastomotic leak was suspected – bile from the drain was noticed. Anastomosis was resected and two layer side-to-side duodenojejunal anastomosis was performed. By then, the pathology report revealed a high grade G3 peripheral/primitive neuroectodermal tumour (PNET)/atypical Ewing sarcoma which expansion to the pancreas (Figs. 3, 4, 5, 6, 7 and 8). R1 resection (at the uncinated process of the pancreas) was confirmed (pT4N1, LVI [2]).

### Tumour histology

An ulcerated tumour in small intestine wall was composed of solid sheets of small blue cells with irregular nuclear contours and prominent nucleoli. Differential diagnosis included poorly differentiated adenocarcinoma, neuroendocrine carcinoma, melanoma, lymphoma, gastrointestinal clear cell sarcoma, synovial sarcoma, rhabdomyosarcoma, epithelioid angiosarcoma, epithelioid sarcoma and germ cell tumour. Immunohistochemical tests were performed and tumour demonstrated diffuse positivity for CD99, ERG, CD56 and focal positivity for Synaptophysin, PanCK, Cam5.2. Tumour was consistently negative for LCA, SMA, Desmin, S100, HMB45, TLE1, Chromogranin A, CDX2, TTF1, PAX8, CK7, CK19, CD31, SALL4, Glucagon, Somatostatin, Gastrin and Serotonin. There was no INI1 loss. Diffuse CD99, ERG, CD56, focal PanCK, Cam5.2 and Synaptophysin immune positivity are characteristic for Ewing sarcoma family of tumours. EWSR1 (22q12) break-apart FISH test and PCR test for EWSR1-ERG, EWSR1-ETV1, EWSR1-FLI1, EWSR1-FEV, EWSR1-ETV4 translocations were performed. Unfortunately, there was no evidence of EWRS1 gene rearrangements by both FISH and PCR EWSR1 methods. Despite this fact there was no evidence of any other tumour and final diagnosis was high grade G3 peripheral/primitive neuroectodermal tumour (PNET)/atypical Ewing’s sarcoma, possibly with a rare translocation.

### Postoperative period

Eleven days after the third surgery bleeding recurred – bleeding from the aorto-mesenteric shunt was found. It was sutured along its wall and intraoperative ultrasound showed reduced diameter of the shunt, and recanalized SMA. During postoperative period due to progressing bile leakage, bile ducts were catheterized trans-hepatically. Nine days later intraabdominal bleeding recurred. Bleeding from the aorta at the shunt area was found and sutured. Small intestine on the other hand showed total necrosis with multiple perforations. The changes were incompatible with life. Nonetheless 48 h later, patient vital signs improved and the condition stabilized. Therefore, during the next surgery necrotised small intestine was removed while closing both ends of the intestines at the demarcation lines and gastrostomy was made for the drainage of the gastric content. During the next few weeks bleeding recurred and multiple (> 50) liver metastases were found. Bleeding was controlled but the patient died because of rapid dissemination of the disease.

## Discussion and Conclusion

Intestinal Ewing’s sarcoma as many other tumours can manifest asymptomatic although there are cases in which it occurs as intussusception [18], perforation [11, 15], intestinal obstruction [14, 23], rupture [4] but usually it presents with abdominal pain or fatigue and weakness caused by mass and/or bleeding as in our case. It can be misdiagnosed as a gynaecologic pathology especially in young females as the literature shows pain can often occur in the lower abdomen [6, 13, 20]. Clinical presentation mainly depends on the affected site of the gastrointestinal tract [19].

Thorough examination should follow CT scan to exclude other acute diseases although there are no specific radiological signs of Ewing’s sarcoma [8]. For this reason Ewing’s sarcoma may initially be treated as GIST [7] as in our case.

To provide proper diagnosis, pathological examination should be carried out. It‘s round cell morphology is similar to neuroblastoma, malignant lymphoma, rhabdomyosarcoma, GIST, and desmoplastic small round cell tumour [14]. While Ewing’s sarcoma shows rosette formation, glycogen deposition, also NSE, S-100 protein, neurosecretory-type granules [12]. In addition CD99 antigen could also be a useful tool [15]. Nowadays molecular testing is a reliable diagnostic method to diagnose ES. The most frequent translocation EWSR1-FLI1 occurs in 85% of cases of ES [2, 22]. Second most common translocation is EWSR1-ERG (5–15%) and in such cases tumour cells are immunohistochemically positive for ERG marker. In our case ERG immunohistochemical reaction was positive, although there was no EWSR1-ERG translocation. However there is a very rare ERG-FUS translocation (< 1%), when tumour cells are also positive for ERG. We suppose that this translocation could occur in our case. It is also mentioned that fusion transcripts should not be the sole criterion of PNET [17].

All Ewing’s sarcoma treatment includes local surgical and/or radiotherapy treatment followed by multidrug systemic chemotherapy [5, 7]. It usually includes vincristine, doxorubicin, cyclophosphamide and dactinomycin [16] although of ifosfamide and etoposide were added. Latter improves outcomes in non-metastatic cases [13].

Prognosis of intestinal Ewing’s sarcoma is not known as there are not enough cases. It could be compared to other sites. Although some authors do not relate tumour size with worse outcomes [13] other find > 5 cm size of the tumour having impact for worse survival [24]. Even in those cases surgical intervention is recommended as it decreases morbidity [21, 25]. Worse survival also may include pelvic site, older patients, poor response to chemotherapy, and metastatic disease at presentation [8] which was in our case. The main two reasons of unfortunate consequence in our case was acute pancreatitis caused by R1 resection at the uncinated process and a very aggressive underlying disease.

To conclude, extraosseus Ewing’s sarcoma is extremely rare entity, with most often poor prognosis.

## Data Availability

Data sharing is not applicable to this article as no datasets were generated or analysed during the current study.

## Abbreviations

ETS: E-twenty six
PNET: Primitive neuroectodermal tumours
pPNET: peripheral primitive neuroectodermal tumour)
PNETs: CNS primitive neuroectodermal tumours
CT: computed tomography
GIST: Gastrointestinal stromal tumour
IMV: inferior mesenteric vein
SMV: superior mesenteric vein
IMA: inferior mesenteric artery
SMA: superior mesenteric artery
LVI: lympho vascular invasion
PCR: polymerase chain reaction
